# A Remote Patient Monitoring Intervention for Patients With Chronic Obstructive Pulmonary Disease and Chronic Heart Failure: Pre-Post Economic Analysis of the Smart Program

**DOI:** 10.2196/10319

**Published:** 2018-12-20

**Authors:** Wanrudee Isaranuwatchai, Olwen Redwood, Adrian Schauer, Tim Van Meer, Jonathan Vallée, Patrick Clifford

**Affiliations:** 1 St. Michael’s Hospital Toronto, ON Canada; 2 Institute of Health Policy, Management and Evaluation University of Toronto Toronto, ON Canada; 3 Canadian Back Institute Toronto, ON Canada; 4 AlayaCare Toronto, ON Canada; 5 Southlake Regional Health Centre Newmarket, ON Canada

**Keywords:** chronic heart failure, chronic obstructive pulmonary disease, costs, economic analysis, emergency department visits, hospitalizations, health service utilization, remote patient monitoring

## Abstract

**Background:**

Exacerbation of chronic obstructive pulmonary disease (COPD) and chronic heart failure (CHF) are associated with high health care costs owing to increased emergency room (ER) visits and hospitalizations. Remote patient monitoring (RPM) interventions aim to improve the monitoring of symptoms to detect early deterioration and provide self-management strategies. As a result, RPM aims to reduce health resource utilization. To date, studies have inconsistently reported the benefits of RPM in chronic illnesses. The Smart Program is an RPM intervention that aims to provide clinical benefit to patients and economic benefit to health care payers.

**Objective:**

This study aims to economically evaluate the potential benefits of the Smart Program in terms of hospitalizations and ER visits and, thus, associated health care costs from the perspective of the public health care system.

**Methods:**

Seventy-four patients diagnosed with COPD or CHF from one hospital site were included in this one-group, pre-post study. The study involved a secondary data analysis of deidentified data collected during the study period – from 3 months before program initiation (baseline), during the program, to 3 months after program completion (follow-up). Descriptive analysis was conducted for the study population characteristics at baseline, the clinical frailty score at baseline and 3-month follow-up, client satisfaction at 3-month follow-up, and number and costs of ER visits and hospitalizations throughout the study period. Furthermore, the cost of the Smart Program over a 3-month period was calculated from the perspective of the potential implementer.

**Results:**

The baseline characteristics of the study population (N=74) showed that the majority of patients had COPD (50/74, 68%), were female (42/74, 57%), and had an average age of 72 (SD 12) years. Using the Wilcoxon signed-rank test, the number of ER visits and hospitalizations, including their associated costs, were significantly reduced between baseline and 3-month follow-up (*P*<.001). The intervention showed a potential 68% and 35% reduction in ER visits and hospitalizations, respectively, between the 3-month pre- and 3-month postintervention period. The average cost of ER visits reduced from Can $243 at baseline to Can $67 during the 3-month follow-up, and reduced from Can $3842 to Can $1399 for hospitalizations.

**Conclusions:**

In this study, the number and cost of ER visits and hospitalizations appeared to be markedly reduced for patients with COPD or CHF when comparing data before and after the Smart Program implementation. Recognizing the limitations of the one-group, pre-post study design, RPM requires an upfront investment, but it has the potential to reduce health care costs to the system over time. This study represents another piece of evidence to support the potential value of RPM among patients with COPD or CHF.

## Introduction

Chronic obstructive pulmonary disease (COPD) and chronic heart failure (CHF) are associated with a high burden (ie, high health care cost) to the system [1,2]. The cost of 1 hospital stay for COPD and CHF in Canada was estimated to be Can $6038 and Can $6222, respectively [3]. Therefore, innovative interventions aimed to reduce the burden on our system (eg, reduce hospitalization) would be beneficial. An example of such interventions is remote patient monitoring (RPM), which aims to provide the “appropriate care at the appropriate time and place in the most appropriate manner” [1], focusing on better disease management [4,5].

A growing amount of literature exists on the potential value of RPM for patients with COPD and CHF; however, the literature has shown both supportive and opposing evidence for RPM [1,6,7]. For example, RPM has been shown to reduce health service utilization and costs (eg, hospitalization and emergency room [ER] visits) among patients with CHF [2,8-12], whereas other studies did not find similar findings [6,7,13,14]. Conversely, some studies reported inconclusive findings [15-17]. Among patients with COPD, evidence was also inconclusive where RPM was found to be an economically attractive option in some studies but not in others [6,18-23]. Furthermore, a number of studies reported the need for more research [24,25].

This study aims to build on and contribute to the current literature by showing a potential value of RPM. Understanding the impact of a health intervention on the cost of hospitalization and ER visits may increase understanding regarding how the intervention will affect the health care system.

The research question was “What was the cost of hospitalization and ER visits of patients receiving RPM over the study period among patients diagnosed with COPD or CHF?” Specifically, based on this one-group, pre-post study design, we aimed to describe the study population and report the use and cost of ER visits and hospitalizations over the study period (from 3 months before program initiation, baseline, to 3 months after program completion, and follow-up) from the perspective of a public health care system in Ontario, Canada.

## Methods

### Study Population and Setting

The study population comprised 74 patients diagnosed with COPD or CHF in 1 hospital site in Toronto, Canada. The inclusion criteria included the following: aged ≥18 years; diagnosed with either COPD or CHF for a minimum of 6 months; ability to communicate in English; and cognitively capable of giving consent. Patients who were unable to provide consent, were a part of a competing program within the same hospital, or had a life expectancy of <6 months were excluded from this study.

This study received a research ethics approval from Southlake Regional Health Centre and St. Michael’s Hospital in Toronto, Ontario, Canada.

### Intervention

The AlayaCare/CBI Smart Program was a collaboration between Southlake Hospital, CBI Health Group, and AlayaCare. The project was conducted between August 2016 and May 2017 on both patients with COPD and CHF to reduce both patient ER visits and hospitalizations. The Smart Program, a type of RPM, was the intervention under study. The RPM is a form of health care that allows patients to use medical devices in the comfort of their home to perform routine tests and send results automatically to their home health care professional. This digital software aims to improve the management of patients’ chronic illness through multisource, self-management techniques, including patient self-identification of symptoms and problem-solving strategies, which will result in the stabilization of their illness status.

### Data Collection and Management

Data were collected by nurses at the point of care through the AlayaCare mobile app or by patients themselves through the AlayaCare RPM app and were stored in the AlayaCare’s secure cloud app. Data were collected at 3 time-points as follows: *baseline* (within 3 months before the program initiation); *during the program*; and *follow-up* (at 3 months after the program completion). During the follow-up period, patients were no longer using the intervention. The deidentified patient-level data were transferred to the research team using encryption and secure internet transmission and used for the economic analysis.

### Variables

The economic analysis conducted in this study was a secondary data analysis that used deidentified data that were collected for the study. Specifically, we descriptively reported patients’ age, sex, medication use, regular medical follow-ups, and the number of patients with, at least, 1 alert for blood pressure, blood oxygen, and weight, including a score from the clinical frailty scale (Table 1).

**Table 1:** The definition of each level on a clinical frailty scale.

Level	Definition
1	Very fit
2	Well
3	Well, with treated comorbidities
4	Apparently vulnerable
5	Mildly frail, some dependence on others for activities of daily living
6	Moderately frail, help needed with instrumental activities of daily living
7	Severely frail

### Health Service Utilization and Cost

Health service utilization data were collected at 3 time-points as follows: *baseline* (within 3 months before the program initiation); *during the program*; and *follow-up* (at 3 months after the program completion). The main types of health service of interest included hospitalization and ER visit, which can be expressed in monetary terms (ie, ER visit cost and hospitalization cost). Subsequently, we converted health service utilization to health care cost using data on health service utilization from the study and standard costing sources for information on the unit cost of hospitalization and ER visit. The unit cost of 1 ER visit was estimated to be Can $159 and was obtained from the Canadian Institute of Health Informatics [26]. A general cost for 1 hospital stay in Ontario was estimated to be Can $5364 [3,27]. Of note, all costs were reported in 2016 Canadian dollars (Can $). Costs from other years were converted to 2016 Can $ using Consumer Price Index under Health Care category published by Statistics Canada [28].

### Statistical Analysis

Descriptive analysis on baseline variables was conducted on age, sex, medication use, regular medical follow-ups, and the number of patients with, at least, 1 alert for blood pressure, blood oxygen, and weight. Descriptive findings on the clinical frailty score were reported at baseline and 3-month follow-up, client satisfaction at 3-month follow-up, and number and costs of ER visits and hospitalizations throughout the study period. The Wilcoxon signed-rank test [29,30] was used to compare the number and cost of ER visits and hospitalizations between baseline and 3-month follow-up, recognizing that the data were from the same individuals. The test focused on the difference in values for each pair of observations. The chosen statistical analysis, the Wilcoxon signed-rank test, adjusted for the nonnormality of health service utilization and cost data [29,30]. Over the study period, the patients’ health service utilization and costs were examined.

In addition, cost description of the program was conducted from the perspective of the potential implementer (eg, the Local Health Integration Network [LHIN]), to report the total cost of delivering the program over a 3-month period. In Ontario, publicly funded health care services are administered on a regional basis by LHINs, which serve as the regional health authority. Each of the 14 LHINs is responsible for a distinct geographical location [31]. The costs associated with delivering the program captured in this study were personnel and supplies and miscellaneous costs.

## Results

### Baseline Characteristics

This study reports descriptive findings on the following: baseline characteristics of the study population; clinical frailty score at baseline and 3-month follow-up; client satisfaction with the intervention at 3-month follow-up; health service utilization that includes the number and costs of ER visits and hospitalizations at baseline, during the program, and follow-up; and cost description of delivering the program over a 3-month period.

Overall, 74 patients were enrolled in the program at baseline. However, at 3-month follow-up, only 67 patients completed the data collection, as 2 people died and 5 were lost to follow-up. The 2 people who died were assessed to be mildly frail with unknown cause of death.

Table 2 reports the baseline characteristics of the study population. The majority of patients had COPD (50/74, 68%). The average age of patients was 72 (SD 12) years, where 42 patients (42/74, 57%) were females. Of all, 60 patients (60/74, 81%) were on, at least, 1 medication, with the number of medications ranging from 1 to 26. For alerts, 29 patients (29/74, 39%) had, at least, 1 weight alert, 68 (68/74, 92%) had, at least, 1 blood pressure alert, and 68 (68/74, 92%) had, at least, 1 blood oxygen alert during the program. Over 85% (64/74) of patients had regular medical follow-ups.

**Table 2:** The baseline characteristics of the study population (N=74).

Variable		Value
Age, mean (SD), range		71.6 (12.0), 44-98
**Sex, n (%)**		
	Female	42 (57)
	Male	32 (43)
**Medications**		
	Had, at least, 1 medication, n (%)	60 (81)
	Number of medications, mean (SD), range	10.0 (5.2), 1-26
Have regular medical follow-ups, n (%)		64 (86)
**Had at least 1 weight alert, n (%), range**		29 (39), 1-29
	1 alert	8
	2 alerts	3
	3-5 alerts	9
	6-10 alerts	6
	10+ alerts	3
**Had at least 1 blood pressure alert, n (%), range**		68 (92), 1-59
	1-5 alerts	24
	6-10 alerts	15
	11-20 alerts	16
	20+ alerts	13
**Had at least 1 blood oxygen alert, n (%), range**		68 (92), 1-89
	1-5 alerts	22
	6-10 alerts	16
	11-20 alerts	14
	20+ alerts	16

### Clinical Frailty

**Figure 1:**
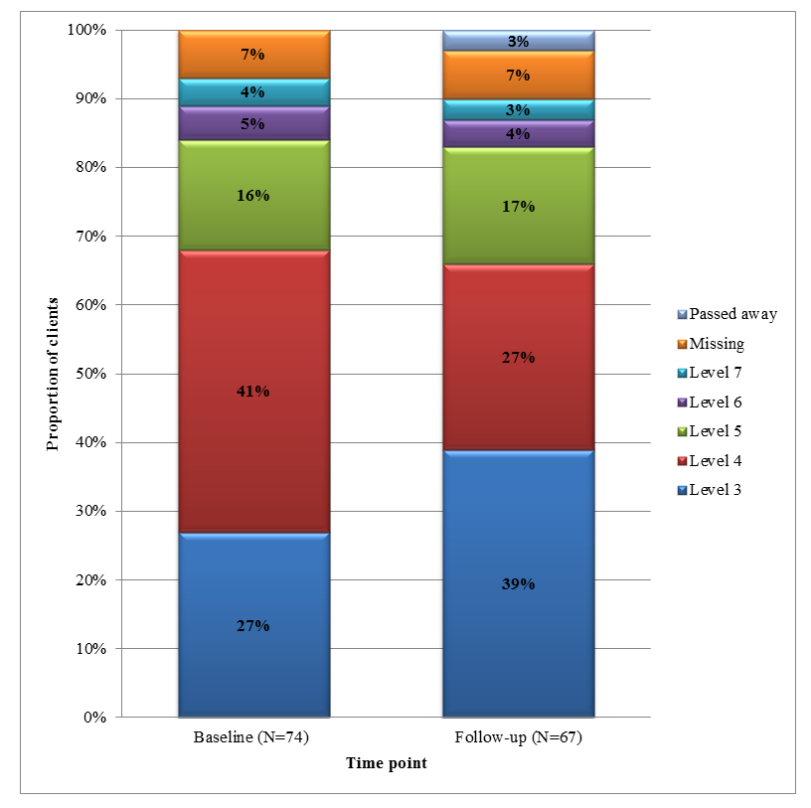
The clinical frailty score at the baseline and 3-month follow-up.

At baseline, the majority of patients (50/74, 68%) reported clinical frailty score to be between 3 (well, with treated comorbidities) and 4 (apparently vulnerable). The level 3 clinical frailty score increased from 27% (20/74) at baseline to 39% (26/67) at 3-month follow-up (Figure 1).

Between the 2 time-points (baseline and follow-up), the majority of patients (49/67, 73%) reported the same score of clinical frailty scale. Approximately 22% (15/67) of patients reported improved score on the frailty scale, while 5% (3/67) reported worsened score.

### Satisfaction

For patient satisfaction with the Smart Program, 91% (61/67) of patients responded at 3-month follow-up. Almost 70% (42/61) of patients strongly agreed that they felt more confident managing their signs and symptoms related to diagnosis. In addition, 97% (59/61) recognized when they should be going to the emergency department, when they could monitor at home, or when they should go to see their physician before a flare-up. Next, 90% (55/61) of patients rated their satisfaction with the Smart Program as very good or excellent, whereas just over 55% (35/61) rated their satisfaction with the use of equipment as very good or excellent. All patients agreed (either somewhat or strongly) that the Smart Program had helped them learn more about their disease, the Smart Program had made a positive difference in their life, and that they would recommend the program to a friend or family member.

### Health Service Utilization

For ER visits, 96% (71/74) of patients had, at least, 1 ER visit during the 3-month period before the program started at baseline; this percentage dropped to 28% (19/67) at 3 months after the program finished. At baseline, exacerbation of chronic disease accounted for the majority of hospitalizations (69%, 51/74) with falls and infections being the other reasons for hospitalization.

During the program, 22% (16/74) of patients had, at least, 1 ER visit, and 9% (7/74) of patients had, at least, 1 hospitalization. The number of ER visits and hospitalizations ranged from 0 to 4 and 0 to 3, respectively. The exacerbation of chronic disease accounted for almost 40% of ER visits, and >70% of hospitalizations. Other reasons included falls and infections.

**Figure 2:**
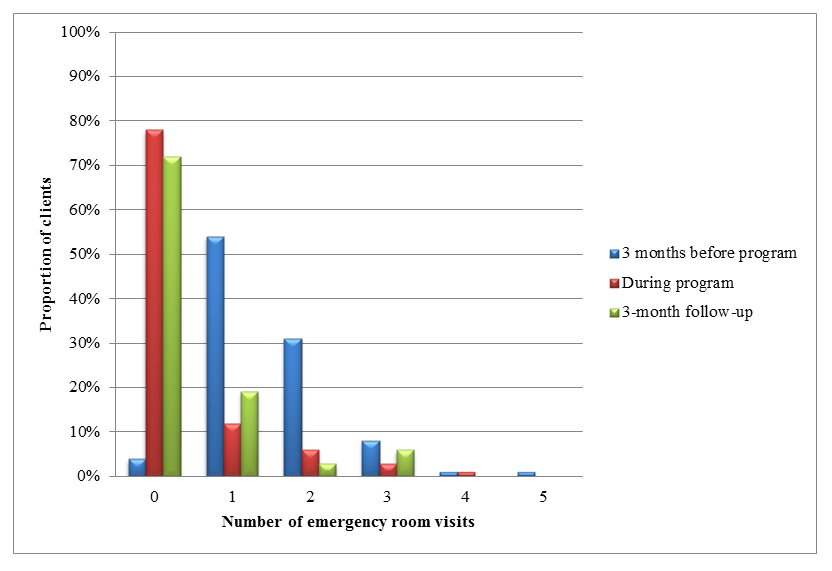
The number of emergency room visits over the study period.

Figure 2 presents the number of ER visits over the study period (N=74 at baseline, ie, 3 months before the program, N=74 during the program, and N=67 at 3-month follow-up). At baseline, the number of visits ranged from 0 to 5, whereas the range was from 0 to 3 at follow-up. At baseline, the majority of patients had, at least, 1 ER visit, whereas the majority of patients had 0 visits during the program and at 3-month follow-up.

**Figure 3:**
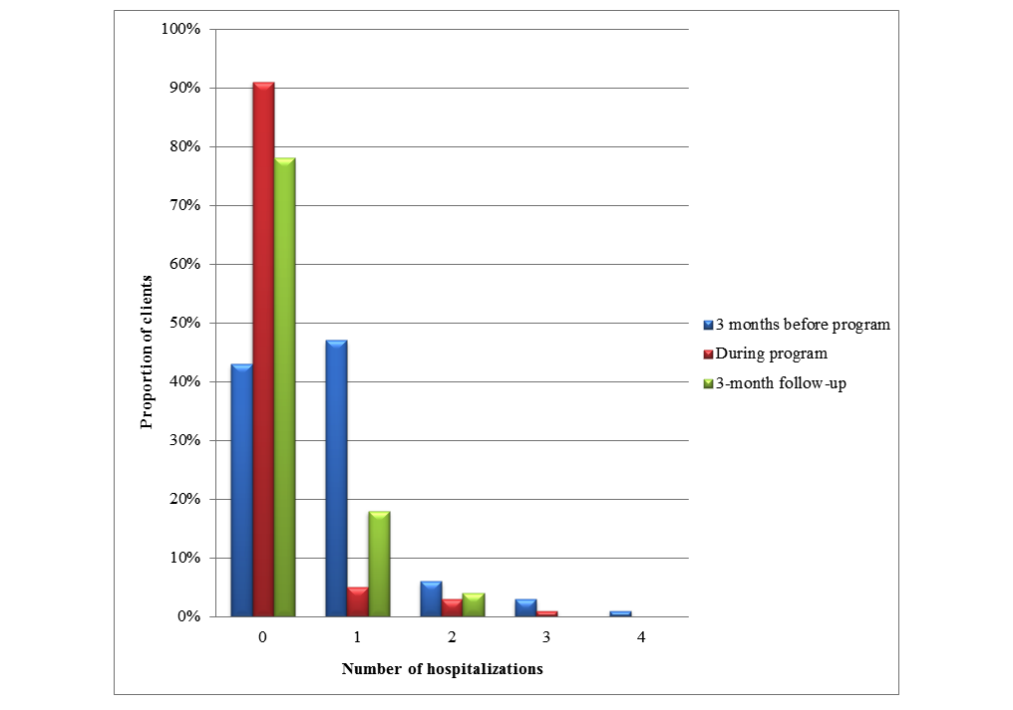
The number of hospitalizations over the study period. ER: emergency room.

Figure 3 presents the number of hospitalizations over the study period. At baseline, the number of hospitalizations ranged from 0 to 4, whereas the range was from 0 to 2 at follow-up. At baseline, the majority of patients (42/74, 57%) had, at least, 1 hospitalization, whereas the majority of patients had 0 hospitalizations during the program and, at 3-month follow-up, only 22% (15/67) of patients had at least 1 hospitalization.

The total number of ER visits and hospitalizations appeared to decline over time in this study population. The number of ER visits and hospitalizations was 71 and 42, respectively, at baseline, and 19 and 15, respectively, at 3-month follow-up.

**Figure 4:**
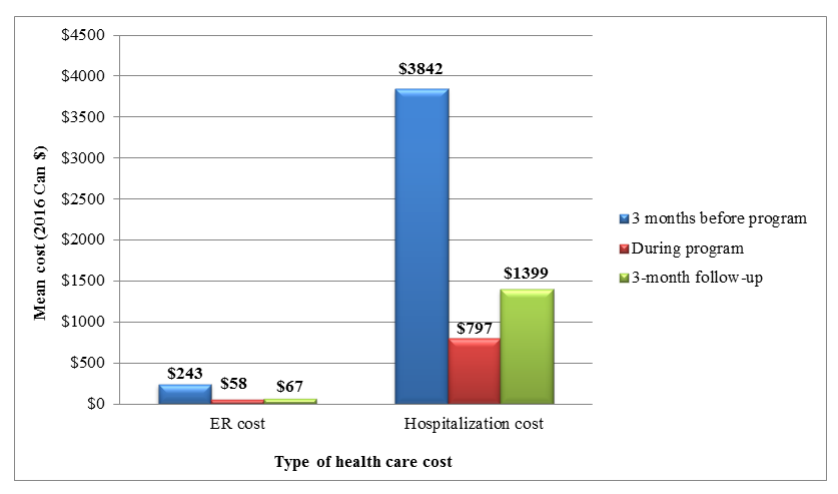
Costs of emergency room visit and hospitalization over the study period.

**Figure 5:**
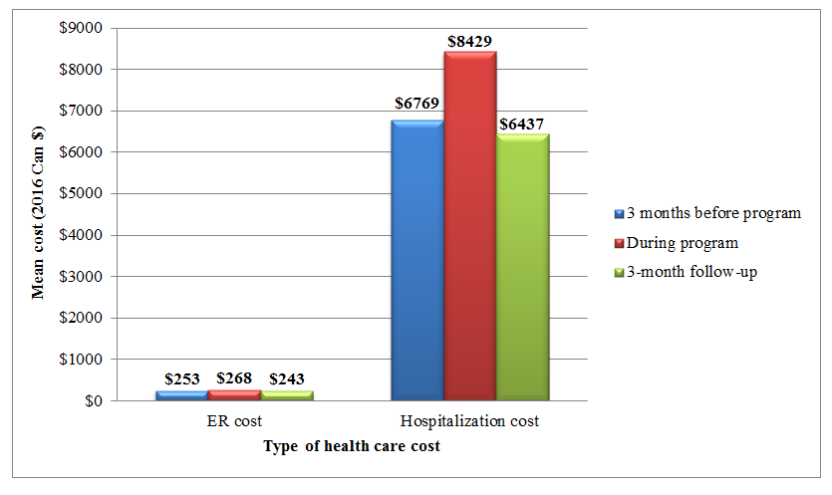
Costs of emergency room visit and hospitalization over the study period among those with at least one visit and hospitalization. ER: emergency room.

Table 3 summarizes the costs of ER visits and hospitalizations over the study period. Between baseline and 3-month follow-up, the number of ER visits and hospitalizations, including their associated costs, was significantly different (*P*<.001) in the direction of lower cost in the follow-up period (Figure 4). Specifically, the average cost for ER visit reduced from Can $243 at baseline (3 months before the program started) to Can $67 during the follow-up (3 months after the program finished; *P*<.001). Similarly, the average hospitalization cost reduced from Can $3842 to Can $1399 (*P*<.001). When considering only patients with, at least, 1 visit, the average costs of ER visit and hospitalization was similar across the 3 time-points (Figure 5).

**Table 3:** Costs of emergency room visit and hospitalization over the study period.

Cost at each time point		Mean cost (SD) in Can $
**At baseline**		
	ER^a^ visit cost	243 (137)
	ER visit cost among users (n=71)	253 (131)
	Hospitalization cost	3842 (4306)
	Hospitalization cost among users (n=42)	6769 (3566)
**During program**		
	ER visit cost	58 (130)
	ER visit cost among users (n=16)	268 (150)
	Hospitalization cost	797 (2763)
	Hospitalization cost among users (n=7)	8429 (4220)
**At 3-month follow-up**		
	ER visit cost	67 (129)
	ER visit cost among users (n=19)	243 (134)
	Hospitalization cost	1399 (2858)
	Hospitalization cost among users (n=15)	6437 (2221)

^a^ER: emergency room.

**Table 4:** Cost components of the Smart Program per patient over a 3-month period.

Cost component	Unit cost in Can $	Number of units	Total cost in Can $
Telehealth nursing cost	34.81/hour	9.4 hours	327.21
Hardware amortized over 3 years	—^a^	—	41.87
Remote patient monitoring software	—	—	105
Case conference (twice per patient)	65	2	130
Wireless data	15/month	3 months	45
Total cost over a 3-month period	—	—	649

^a^Not applicable.

### Cost Description

Over a 3-month period, the total cost to deliver the program was Can $649 per patient; this amount accounted for both personnel and supplies and miscellaneous costs. Personnel costs comprised salary and benefits for a telehealth nurse. Supplies and miscellaneous costs included hardware, RPM software, case conferences (2 times per patient), and wireless data. Table 4 presents the cost of providing the Smart Program over a 3-month period.

## Discussion

### Principal Findings

This study analyzed the baseline characteristics of the study population and examined the number and cost of ER visits and hospitalizations over the study period from the perspective of a public health care system in Ontario, Canada. Of 74 patients included in this study, a majority had COPD (50/74, 68%), were female (42/74, 57%), and had an average age of 72 (SD 12) years. Approximately 80% (60/74) were on, at least, 1 medication, and >85% (64/74) of patients had regular medical follow-ups. For alerts, 39% (29/74) had, at least, 1 weight alert, 92% (68/74) had, at least, 1 blood pressure alert, and 92% (68/74) had, at least, 1 blood oxygen alert during the program. The proportion of patients with the clinical frailty score of 3 (well, with treated comorbidities) also increased from 27% (20/74) at baseline to 39% (26/67) during the 3-month follow-up period, which showed favorable outcomes with the use of the Smart Program.

Among patients diagnosed with a chronic illness of COPD or CHF, the number and cost of ER visits and hospitalizations appeared to be markedly reduced when compared between the 3-month period before the program started and the 3-month period after the program finished. The average cost of ER visit reduced from Can $243 at baseline to Can $67 at follow-up, and reduced from Can $3842 to Can $1399 for hospitalizations. These reductions were partly attributed to the findings that the number of ER visits and hospitalizations reduced, while this intervention costs approximately Can $649 to implement for 1 patient over a 3-month period. Notably, when considering only patients with, at least, 1 visit, the average costs of ER visit and hospitalization were similar across the 3 time-points.

The RPM literature provides both supportive and opposing evidence to verify the value of RPM. For example, a reduction in direct health care cost was found in a review by Seto to be between 1.6% and 68.3% [2], and in a study by Scalvini et al to be approximately 10% [10]. This study reports the reduction of hospitalization to be 35% and of ER visits to be 68%. These differences could be attributed to a number of factors such as target population, the range of supports provided as part of the RPM, and settings.

### Strengths and Limitations

This study has strengths and limitations. As the literature has recommended RPM that can support more than one condition [32], this study shows that an RPM system targeting more than one condition can be successfully implemented. A review [33] suggested that more details on cost, including amortization, should be made explicit as we have done here. This study represents a case study to support the potential value of RPM by examining both costs and outcomes of RPM where the outcomes (measured in hospitalization and ER visits) have been converted to monetary values.

Given the nature of the study design (one-group, pre-post study design), the findings contributed only to the trends of health service utilization and cost over the study period. Future research could build on this work and design a study with a comparator group to comprehensively examine the potential impact of RPM in the study population. In addition, future research could explore the options to conduct the analysis with a longer follow-up time from another perspective, which could include other costs (eg, costs to patients and caregivers, which has been suggested to be an important element [34]), and other outcomes such as the quality of life and productivity loss. Furthermore, a subgroup analysis (eg, patients with comorbidities) could be explored to validate the impact of RPM.

### Conclusions

In summary, RPM (in this case, the Smart Program) may require upfront investment but it has the potential to reduce health care costs to the system over time. This study represents a piece of evidence to support the potential value of RPM among patients with COPD or CHF. This intervention shows a potential 68% reduction in ER visits and a 35% reduction in hospitalizations between the 3-month pre- and 3-month postintervention period. Recognizing the limitations of the one-group, pre-post study design, RPM could be an economically attractive option to explore for a health system in savings from reductions in ER visits and hospitalizations among patients with COPD or CHF.

## Abbreviations

CHF: chronic heart failure
COPD: chronic obstructive pulmonary disease
ER: emergency room
LHIN: Local Health Integration Network
RPM: remote patient monitoring
